# Dataset of theoretical Molecular Electrostatic Potential (MEP), Highest Occupied Molecular Orbital-Lowest Unoccupied Molecular Orbital (HOMO-LUMO) band gap and experimental cole-cole plot of 4-(*ortho-, meta-* and *para-*fluorophenyl)thiosemicarbazide isomers

**DOI:** 10.1016/j.dib.2020.106299

**Published:** 2020-09-09

**Authors:** Sharmili Silvarajoo, Uwaisulqarni M. Osman, Khadijah H. Kamarudin, Mohd Hasmizam Razali, Hanis Mohd Yusoff, Irshad Ul Haq Bhat, Mohd Zul Helmi Rozaini, Yusnita Juahir

**Affiliations:** a Faculty of Science and Marine Environment, Universiti Malaysia Terengganu, 21030 Kuala Nerus, Terengganu, Malaysia; b Advanced Nano Materials Research Group (ANOMA), Ionic State Analysis (ISA) Laboratory, Universiti Malaysia Terengganu, 21030 Kuala Nerus, Terengganu, Malaysia; c Institute of Biotechnology Marine, Universiti Malaysia Terengganu, 21030 Kuala Nerus, Terengganu, Malaysia; d Department of Chemistry, Faculty of Science and Mathematics, Universiti Pendidikan Sultan Idris, 35900 Tanjong Malim, Perak, Malaysia

**Keywords:** Thiosemicarbazide, DFT, Isomers, Cole-cole plot, Conductivity

## Abstract

One-pot synthetic method was adopted to prepare three isomers 4-(*ortho-*fluorophenyl)thiosemi- carbazide), 4-(*meta-*fluorophenyl)thiosemicarbazide and 4-(*para-*fluorophenyl)thiosemicarbazide. The products were obtained in ethanolic solution from a reaction between *ortho, meta* and *para* derivatives of fluorophenyl isothiocyanate and hydrazine hydrate. This work presents the theoretical Molecular Electrostatic Potential (MEP) and Highest Occupied Molecular Orbital-Lowest Unoccupied Molecular Orbital (HOMO-LUMO) computational data through Gaussview 5.0.9 and Gaussian09 software. Experimental Cole-cole plot for conductivity determination was also illustrated. The present data is important to manipulate the properties of compounds according to the position of a fluorine atom.

## Specifications Table

**Table:** 

Subject	Chemistry
Specific subject area	Theoretical chemistry, Material Science
Type of data	Table, image, graph and figure
How data were acquired	Both HOMO-LUMO and MEP computational data was obtained through GaussView 5.0.9 and Gaussian 09 software; The Cole-cole plot was measured by Electrical Impedance Spectroscopy (EIS) using HIOKI 3532-50 LCR Hi-Tester interfaced to a computer.
Data format	Raw Analyzed
Parameters for data collection	Theoretical computational data was carried out through 6–311G (d,p) basic set with B3LYP DFT method. The Cole-cole plot was measured at room temperature (303 K) in a frequency range between 50 Hz to 1M Hz.
Description of data collection	The GaussView 5.0.9 and Gaussian 09 software were carried out using typical personal computer. The Cole-cole plot data was collected *via* the raw files from the respective instruments.
Data source location	Universiti Malaysia Terengganu, 21030 Kuala Nerus, Terengganu, Malaysia.
Data accessibility	Repository name: Mendeley Data identification number: 10.17632/94ffhf2224.1 Direct URL to data: https://data.mendeley.com/datasets/94ffhf2224/1

## Value of the Data

• The obtained data is useful to researchers who are developing a chemical database that is specifically related to thiomisecarbazide derivatives.
• The correlation between theoretical MEP, HOMO-LUMO computational data and experimental Cole-cole plot are important to produce potential thiosemicarbazide derivatives used as polymer electrolytes.
• The data provides measurements of isomers with variety of different fluorine atom positions which allows reader to design the properties of compounds accordingly.

## 1 Data Description

**Fig. 1:**
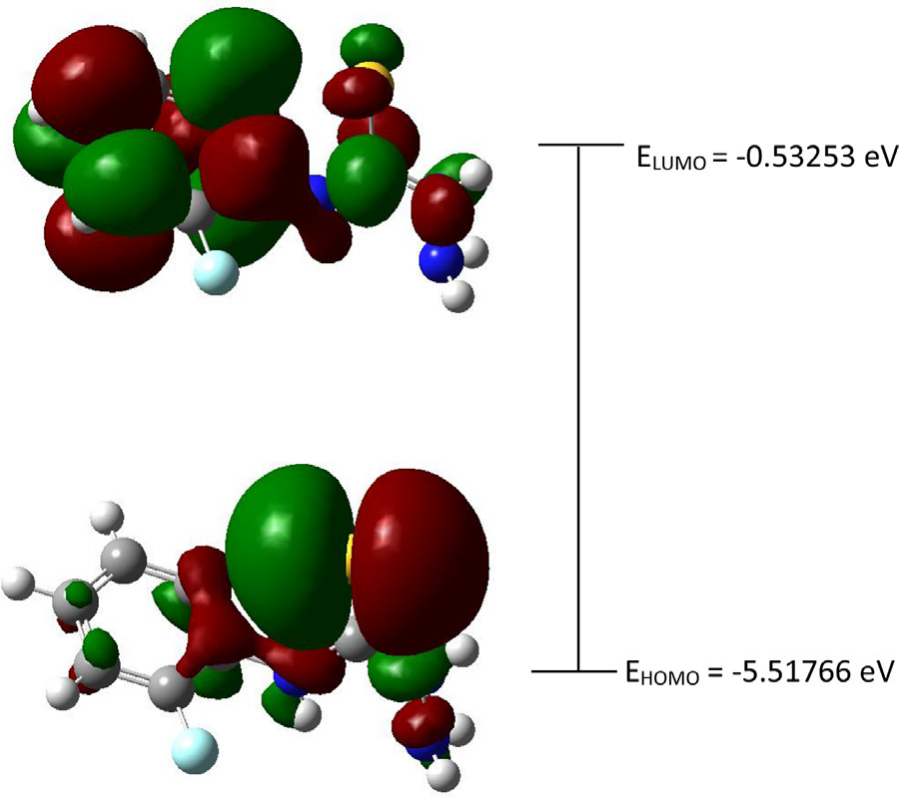
The Highest Occupied Molecular Orbital (HOMO) and Lowest Unoccupied Molecular Orbital (LUMO) for the 4-(*ortho-*fluorophenyl)thiosemicarbazide (In ground state) (isovalue = 0.02).

**Fig. 2:**
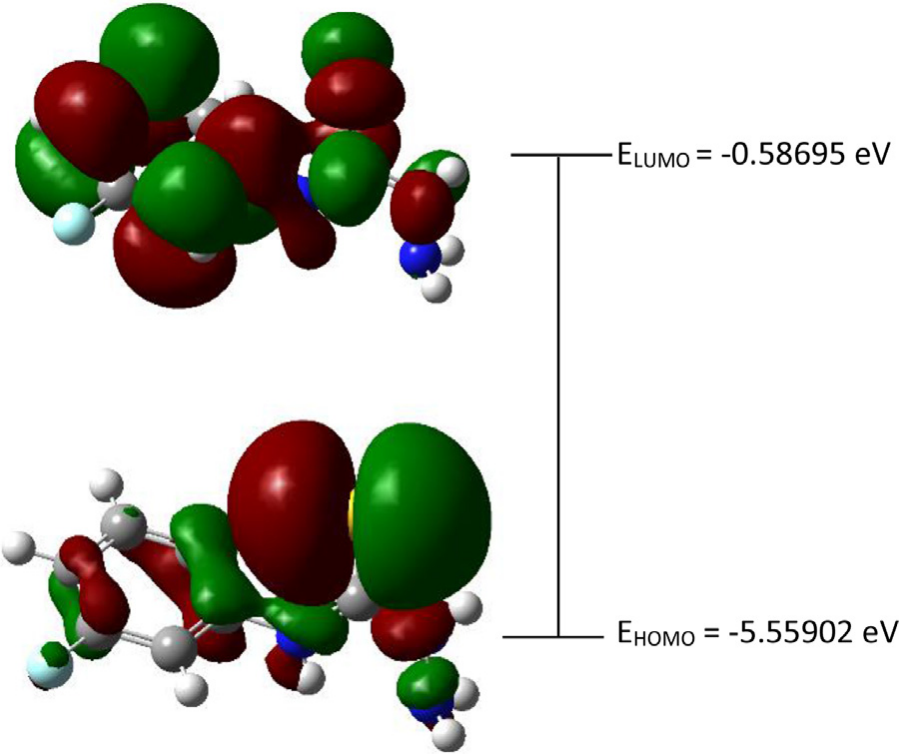
The Highest Occupied Molecular Orbital (HOMO) and Lowest Unoccupied Molecular Orbital (LUMO) for the 4-(*meta-*fluorophenyl)thiosemicarbazide (In ground state) (isovalue = 0.02).

**Fig. 3:**
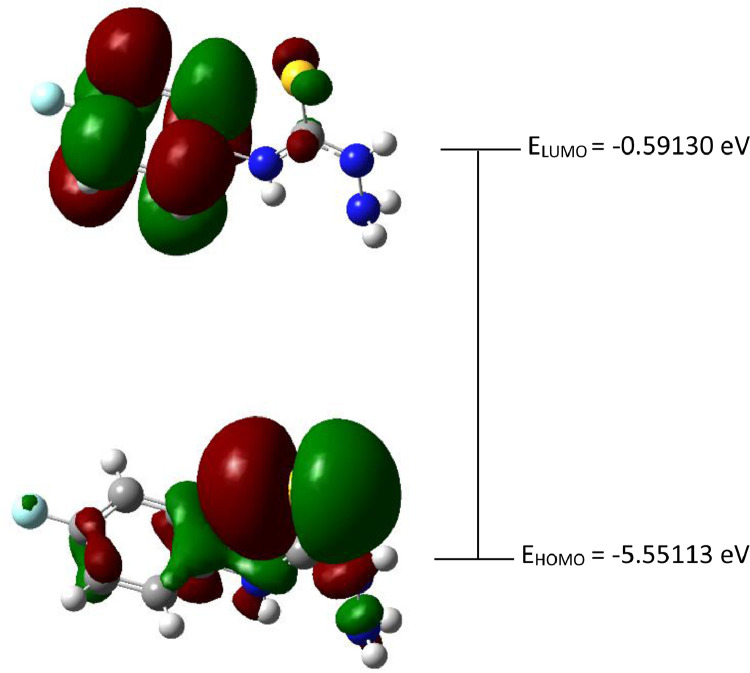
The Highest Occupied Molecular Orbital (HOMO) and Lowest Unoccupied Molecular Orbital (LUMO) for the 4-(*meta-*fluorophenyl)thiosemicarbazide (In ground state) (isovalue = 0.02).

**Fig. 4:**
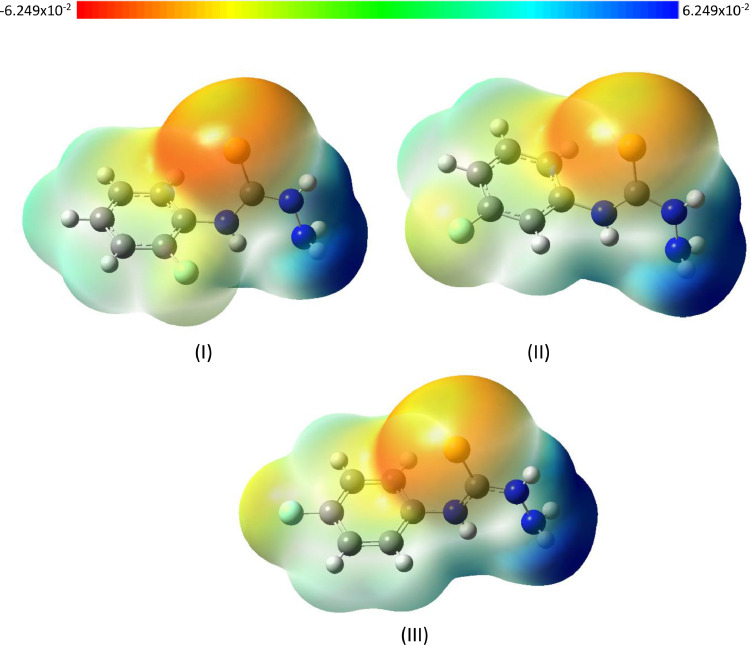
Molecular Electrostatic Potential (MEP) surface diagram of (I) 4-(*ortho-*fluorophenyl) thiosemicarbazide (II) 4-(*meta-*fluorophenyl)thiosemicarbazide and (III) 4-(*para-*fluorophenyl) thiosemicarbazide isomers

**Fig. 5:**
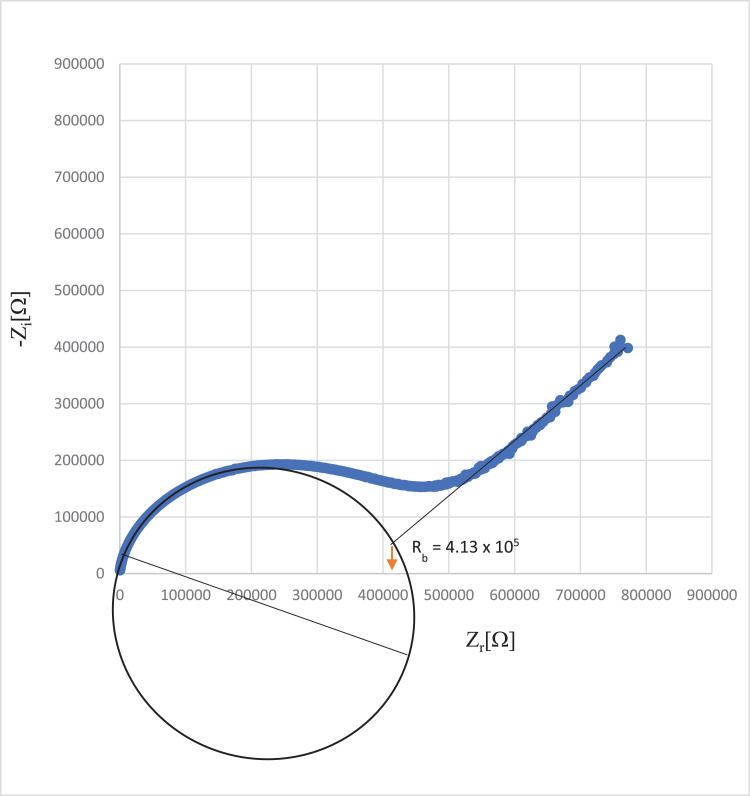
Cole-cole plot of 4-(*ortho-*fluorophenyl)thiosemicarbazide thin film

**Fig. 6:**
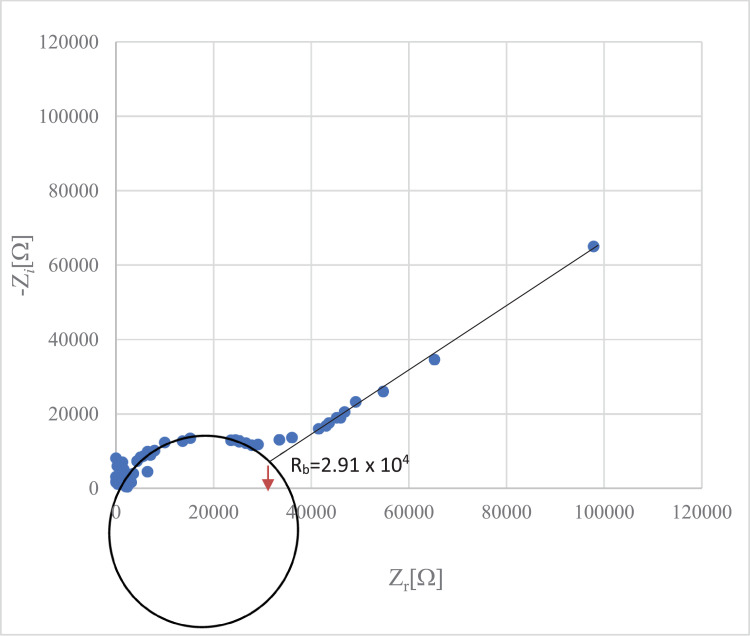
Cole-cole plot of 4-(*mta-*fluorophenyl)thiosemicarbazide thin film

**Fig. 7:**
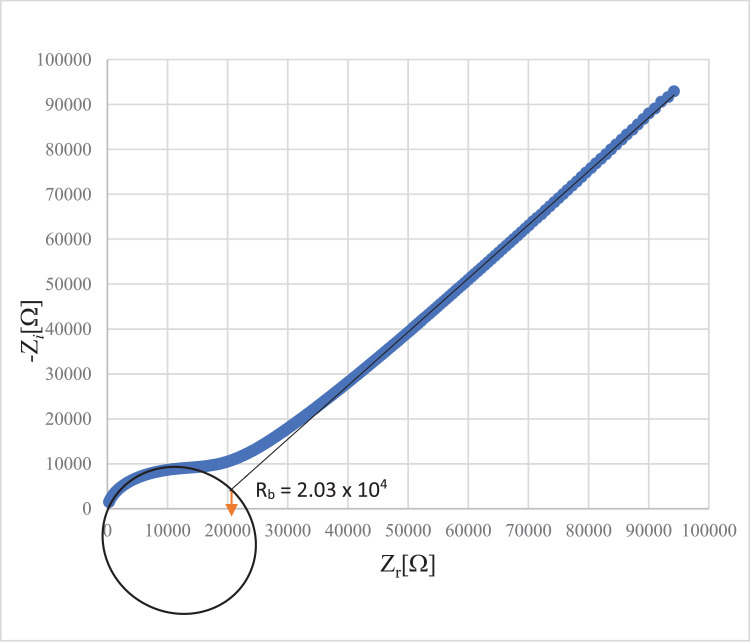
Cole-cole plot of 4-(*para-*fluorophenyl)thiosemicarbazide thin film

Generally, all present data was related with previous reported on crystal structure of 4-(*para-*fluorophenyl)thiosemicarbazide [1] with CCDC number: 1003473 (https://www.ccdc.cam.ac.uk/structures/Search?Ccdcid=1003473&DatabaseToSearch=Published). Theoretical computational data for both Highest Occupied Molecular Orbital-Lowest Unoccupied Molecular Orbital (HOMO-LUMO) and Molecular Electrostatic Potential (MEP) are presented in Fig. 1, Fig. 2, Fig. 3, Fig. 4, respectively. Whereas, in Table 1 calculated data derived from energy gap values using similar equation was reported [2,3]. The experimental cole-cole plot for conductive interpretation if given in Fig. 5, Fig. 6, Fig. 7. The calculated conductivity values obtained from the Cole-cole plot were summarized in Table 2.

**Table 1:** Calculated *E*_gap_, chemical hardness (*η*) softness (*σ*) and electronegativity (*χ*) of presence isomers.

Isomers	*E*_gap_ (eV)	*η* (eV)	*σ* (eV)	*χ* (eV)
4-(*ortho-*fluorophenyl)thiosemicarbazide	4.98513	2.49257	0.40119	−2.49570
4-(*meta-*fluorophenyl)thiosemicarbazide	4.97207	2.48604	0.40225	−2.48604
4-(*para-*fluorophenyl)thiosemicarbazide	4.95983	2.47992	0.40324	−2.47992

**Table 2:** Thickness, bulk resistance and conductivity of the polymer electrolyte films.

Isomers	*R_b_*	Thickness, *t* (cm)	Conductivity, *σ* (S/cm)
4-(*ortho-*fluorophenyl)thiosemicarbazide	4.13 × 10^5^	0.0144	1.11 × 10^−8^
4-(*meta-*fluorophenyl)thiosemicarbazide	2.91 × 10^4^	0.0017	1.86 × 10^−8^
4-(*para-*fluorophenyl)thiosemicarbazide	2.03 × 10^4^	0.0039	7.21 × 10^−8^

## 2 Experimental Design, Material, and Methods

### 2.1 Material

All chemicals and solvents were of analytical grade and were used as supplied.

### 2.2 Preparation of 4-(ortho-, meta- and para-fluorophenyl)thiosemicarbazide isomers

For 4-(*ortho*-fluorophenyl)thiosemicarbazide compound, a suspension of *ortho*-fluorophenyl isothiocyanate (1.53 g, 0.01 mol) with hydrazine hydrate (0.320 ml, 0.01 mol) was refluxed in ethanol (50 ml) for 5 h. The white precipitate formed was filtered and washed with cold ethanol. Finally, the precipitate was recrystallized from hot ethanol, dried and stored in desiccator filled with silica gel.

Whereas, for both 4-(*meta*-fluorophenyl)thiosemicarbazide and 4-(*para*-fluorophenyl) thiosemicarbazide compounds, *meta*-fluorophenyl isothiocyanate and *para*-fluorophenyl isothiocyanate reactants were used instead of *ortho*-fluorophenyl isothiocyanate.

### 2.3 Computational details

Optimized structure of all isomers were carried out with GaussView 5.0.9 and Gaussian 09 software package programme [4]. In theoretical studies, 6–311G (*d,p*) was selected as basic set due to standard theory level for C, H, N, S and F elements. Furthermore, the Density Functional Theory (DFT) method, named Becke, 3-parameter, Lee-Yang-Parr (B3LYP) was selected as method to interpret Highest Occupied Molecular Orbital (HOMO) and Lowest Unoccupied Molecular Orbital (LUMO) analysis in their optimized structures [5, 6]. The structure optimization was obtained at the minimum potential energy. Thus, all theoretical parameters were calculated at the minimum energy optimization.

The red and green colour of the orbital represents the positive and negative phase accordingly. HOMO-LUMO determination and its other several important key factors for conductivity activity which like energy gap (Δ*E*_gap_), hardness (*η*), softness (*σ*) and the global electronegativity (*χ*) were calculated by using Eqs. (1)–(4) [2,3].

(1) $$\Delta E_{gap}=E_{LUMO}-E_{HOMO}$$

(2) $$\eta =\frac{E_{LUMO}-E_{HOMO}}{2}$$

(3) σ=1/η

(4) $$\chi =-\frac{E_{LUMO}-E_{HOMO}}{2}$$

Molecular Electrostatic Potential (MEP) is useful to visualize both yellow and blue regions indicate the electrophilic and nucleophilic regions, respectively.

### 2.4 Polymer electrolyte film preparation

In a beaker, 1.0 g of carboxymethyl cellulose (CMC) was added to 20 mL of distilled water and stirred until it was completely dissolved. In the meantime, for a separate solution containing 0.381 g (16 wt%) of 4-(*ortho*-fluorophenyl)thiosemicarbazide dissolved in 30 mL of ethanol was added dropwise into the CMC solution. The mixture was left stirring until a homogeneous solution formed. Propylene carbonate (8 wt%) was then added into the mixture. The mixture was casted into the petri dishes and dried in the oven at 60 °C for 14 h to form a thin film. The same procedure was applied for the polymer electrolyte films containing 4-(*meta*-fluorophenyl)thiosemicarbazide and 4-(*para*-fluorophenyl)thiosemi- carbazide isomers.

### 2.5 Electrochemical impedance spectroscopy

The polymer electrolyte films were cut into small discs of 2 cm diameter and sandwiched between two stainless steel electrodes under spring pressure. The measurements were carried out at room temperature (303 K). The conductivity of the PE films was calculated as in Eq. (5) [7,8].

(5) $$\sigma =t/A\times R_{b}$$

where: *t* = thickness of PE film (cm)

*A* = surface contact area of PE film (cm^2^)

*R_b_* = bulk resistance of PE film (Ω)

## Declaration of Competing Interest

The authors declare that they have no known competing financial interests or personal relationships which have, or could be perceived to have, influenced the work reported in this article.

## Appendix Supplementary materials

Image, application 1
